# Identification and RNA Interference-Based Functional Analysis of Chitin Deacetylase Genes in *Bemisia tabaci*

**DOI:** 10.3390/insects17060628

**Published:** 2026-06-15

**Authors:** Dejun Kong, Yangnan Hou, Yijing Xiong, Yu Wang, Jigang Li

**Affiliations:** 1 School of Life Sciences, Hebei University, Baoding 071002, China; xk143285@163.com (D.K.); hynn145890@163.com (Y.H.); xiongyijing0314@163.com (Y.X.); wangyu990312@163.com (Y.W.); 2 Key Laboratory of Microbial Diversity Research and Application of Hebei Province, Hebei University, Baoding 071002, China; 3 Engineering Research Center of Ecological Safety and Conservation in Beijing-Tianjin-Hebei (Xiong’an New Area) of MOE, Hebei University, Baoding 071002, China

**Keywords:** *Bemisia tabaci*, chitin deacetylase, RNA interference, nanocarrier, transgenic plant, pest control

## Abstract

Chitin deacetylases (CDAs) play important roles in the growth and development of insects. In this study, four genes encoding chitin deacetylases (BtCDAs) were identified and characterized in the genome of the whitefly *Bemisia tabaci* MEAM1 cryptic species through bioinformatic annotation. Phylogenetic analysis showed that insect chitin deacetylases could be divided into five groups, with no Group II, IV, or V CDAs found in *B. tabaci*. Investigation of the developmental expression patterns of the four *BtCDAs* revealed that *BtCDA1*, *BtCDA2a*, *BtCDA2b*, and *BtCDA4* were expressed at varying levels during the egg and nymph stages, with extremely low expression levels in adults. Delivery of dsRNA targeting *BtCDA1*, *BtCDA2a/b*, and *BtCDA4* to fourth-instar nymphs of *B. tabaci* using the nanomaterial SPc resulted in significant gene silencing and mortality. A fusion gene of the three *BtCDAs* was designed based on the four BtCDA genes and subjected to RNAi experiments, demonstrating that both transgenic tomato and SPc-mediated delivery of fusion gene dsRNA could silence all *BtCDA* genes. These preliminary results indicate that the RNAi targeting of *BtCDAs* leads to substantial mortality in *B. tabaci*, highlighting the potential of *BtCDAs* as effective targets for RNAi-based pest management strategies.

## Simple Summary

The whitefly, *Bemisia tabaci*, is a globally destructive agricultural pest that causes substantial economic losses through direct plant sap feeding and transmission of plant viruses. Current management relies primarily on chemical insecticides. RNA interference-based insect pest control is considered a green and sustainable alternative *B. tabaci* control strategy. In this study, we identified four key chitin deacetylase genes in *B. tabaci*—*BtCDA1*, *BtCDA2a*, *BtCDA2b*, and *BtCDA4*—that are involved in the formation of critical structures, such as the insect cuticle and midgut matrix. Delivery of corresponding double-stranded RNAs (dsRNAs) into *B. tabaci* using the nanomaterial SPc, or allowing whiteflies to feed on transgenic tomato plants producing dsRNAs, effectively silenced the target genes. These findings demonstrate that RNA interference-mediated targeting of these genes is promising for developing novel, environmentally friendly strategies for *B. tabaci* management.

## 1. Introduction

*Bemisia tabaci* (Hemiptera: Aleyrodidae) is a globally important agricultural pest with a host range including various ornamental plants, vegetables, some fiber crops, and grain legumes [1]. *B. tabaci* possesses piercing-sucking mouthparts for extracting nutrients from plant phloem and transmits numerous plant viruses [2]. Additionally, *B. tabaci* excretes sticky honeydew, which interferes with plant photosynthesis and thereby reduces crop economic value [3]. Studies have shown that *B. tabaci* comprises multiple cryptic species, among which Mediterranean (MED, or Q biotype) and Middle East-Asia Minor 1 (MEAM1, or B biotype) are the most invasive and destructive [1,4,5]. Currently, with the growing concern about issues arising from the overuse of chemical insecticides for controlling *B. tabaci* [6,7,8], RNA interference has been considered as a promising alternative approach for pest control [9]. It is a post-transcriptional gene silencing mechanism whereby the introduction of dsRNA into cells leads to the degradation of specific mRNA, resulting in gene functional defects and even death in insects [10,11,12]. The functionality of RNAi has now been well established in various insects. For example, dsRNA designed against the inhibitor of apoptosis (IAP) gene in the tarnished plant bug (*Lygus lineolaris*), delivered via microinjection, significantly shortened the lifespan of this pest [13]. Feeding dsRNA targeting the trehalose phosphate synthase (TPS) gene to the brown planthopper (*Nilaparvata lugens*) significantly reduced TPS gene expression levels in this insect [14]. As we have reported previously, nanoparticle-mediated dsRNA delivery achieved efficient RNA interference of chitin metabolism genes in *B. tabaci*, causing increased nymph mortality and adult emergence failure [15].

Chitin is a linear polymer composed of numerous N-acetylglucosamine units linked by β-1,4-glycosidic bonds and represents one of the most important biopolymers in nature [16]. In insects, chitin serves as a structural component of extracellular matrices, including the exoskeletal cuticle and the peritrophic matrix (PM) of the midgut [17,18,19]. Insect growth and development are strictly dependent on the ability to remodel chitin structures [20]. Consequently, insects continuously synthesize and degrade chitin in a highly regulated manner to facilitate molting and peritrophic matrix renewal [21]. Aberrations in chitin metabolism can result in molting defects and even mortality [16,22].

Chitin deacetylases (CDAs) are key enzymes involved in chitin modification, belonging to carbohydrate esterase family 4 (CE4). Their catalytic function involves hydrolyzing acetyl groups from N-acetylglucosamine residues in chitin molecules to produce chitosan [23]. CDAs are widely distributed in bacteria, fungi, and insects. Notably, this enzyme is highly conserved in insects [24]. The first insect CDA protein was identified by Guo et al. from the midgut of cabbage looper (*Trichoplusia ni*) [25]. In recent years, with the increasing availability of genomic and transcriptomic data, CDA gene families in various insects have been systematically identified and functionally characterized [26,27,28,29,30,31]. Studies have shown that insect CDA genes are typically divided into five groups (I–V), with distinct differences in domain composition, expression patterns, and biological functions among members of different groups [31]. Group I CDA members include CDA1 and CDA2, while Group II consists of CDA3. These two groups of CDAs possess a chitin-binding peritrophin-A domain (CBD), a polysaccharide deacetylase-like catalytic domain (CE4), and a low-density lipoprotein receptor class A domain (LDLa). Group III and IV CDA members are CDA4 and CDA5, respectively. These CDAs retain the CBD and CE4 domains but lack the LDLa domain. Group V contains only a CE4 domain, with members including CDA6, CDA7, CDA8, and CDA9 [23]. In the red flour beetle (*Tribolium castaneum*), RNAi of Group I CDAs resulted in larval molting failure, whereas interference of Group III CDAs affected peritrophic matrix integrity and increased susceptibility to pathogens [32]. In hemipteran pests such as the brown planthopper (*N. lugens*) and the white-backed planthopper (*Sogatella furcifera*), CDA genes have also been demonstrated to affect nymph molting and survival, showing potential as RNA interference targets [28,29].

Among the diverse nanocarriers developed for dsRNA delivery, star polycation (SPc) emerges as a particularly promising candidate. It combines high structural stability, effective protection of dsRNA from nuclease degradation, efficient cellular uptake, and negligible toxicity to host plants and the environment. Recent studies, including our previous work [33], have demonstrated the utility of SPc as an efficient RNAi delivery system for gene function analysis in tiny insects like *B. tabaci* [34].

In this study, we used bioinformatic tools to make a whole-genome identification of chitin deacetylases (BtCDAs) in the *B. tabaci* genome. The molecular phylogenic relationship of BtCDAs with CDAs from other insect species was analyzed. Based on the temporal expression patterns of these genes, we performed RNA interference of *BtCDAs* on *B. tabaci* nymphs to evaluate their roles in *B. tabaci* growth and development, with the aim of providing novel targets and theoretical reference for RNAi-based *B. tabaci* control strategies.

## 2. Materials and Methods

### 2.1. Insect Rearing

Adults of the MEAM1 cryptic species of *B. tabaci* were collected and identified from the campus of Hebei University (Wusi East Road Campus, Baoding, Hebei Province, China) in October 2022. Subsequently, the *B. tabaci* strain has been maintained continuously on cotton (*Gossypium hirsutum*) seedlings in insect-proof cages. The rearing conditions were as follows: temperature 26 ± 1 °C, relative humidity (RH) 60 ± 10%, and a photoperiod of 16 h light and 8 h dark. Every 3 to 5 generations, the mitochondrial cytochrome oxidase I (mtDNA COI) gene sequence was amplified using polymerase chain reaction (PCR) and sequenced to monitor the strain purity [35].

### 2.2. Identification and Molecular Cloning of B.tabaci CDA Genes

The genome data of *B. tabaci* MEAM1 cryptic species were retrieved from the NCBI database (https://www.ncbi.nlm.nih.gov/datasets/genome/GCF_001854935.1/, accessed on 1 September 2024). Insect CDA protein sequences were downloaded from NCBI Entrez (https://www.ncbi.nlm.nih.gov/, accessed on 1 September 2025). The domains of these sequences were annotated using InterProScan software (Version 5.76-107.0) with CDD, Pfam, and SignalP_EUK programs [36]. For curation of the insect CDA protein sequences, proteins containing domains other than LDLa (cd00112 in CDD database and PF00057 in Pfam database), chitin deacetylase (cd10974 and cd10975 in CDD database and PF01522 in Pfam database), chitin binding (PF01607 in Pfam database), and signal peptides (SignalP-noTM in SignalP_EUK database) were excluded from the dataset. A total of 1000 randomly selected insect CDA sequences were aligned using the MAFFT7 multiple sequence alignment tool [37]. The hmmbuild program in the HMMER3.4 software package was used to generate a profile hidden Markov model (HMM) from the alignment file, which was then employed to search the *B. tabaci* proteome data using hmmsearch program in the same software package to obtain *B. tabaci* CDA candidate sequences [38].

### 2.3. Bioinformatic Analysis of BtCDAs

The molecular weight, isoelectric point, and hydrophilicity/hydrophobicity of the candidates BtCDAs were predicted using the ExPASy Proteomics Server (http://expasy.org/, accessed on 1 November 2025). Subcellular localization was predicted using WoLF PSORT II (https://www.genscript.com/wolf-psort.html, accessed on 15 October 2025). Signal peptides were predicted using SignalP 6.0 (https://services.healthtech.dtu.dk/services/SignalP-6.0/, accessed on 15 October 2025). Protein domains of candidate BtCDA sequences were annotated using InterProScan software (Version 5.76-107.0) with CDD, Pfam, and SignalP_EUK programs [36]. Phylogenetic trees were constructed using MEGA 11 software with the neighbor-joining (NJ) method and 1000 bootstrap replicates [39]. Protein domain architectures and phylogenetic trees were visualized using TBtools (Version 2.146) [40].

### 2.4. Gene Cloning Using RT-PCR

*B. tabaci* total RNA was extracted using a TRIzol Reagent (Invitrogen, Carlsbad, CA, USA) according to the manufacturer’s instructions. The concentration and integrity of RNA were checked using a spectrophotometer (BIO-DL, Shanghai, China) and 1% agarose gel electrophoresis. To synthesize the first-strand cDNA and amplify the cDNA sequences of *BtCDAs*, a HiFiScript gDNA Removal RT MasterMix kit (CWBIO, Taizhou, China) was used, and a PrimeSTAR Max DNA Polymerase (Takara, Dalian, China) was employed. Primers used are listed in Supplementary Table S1. The PCR products were ligated with a pTOPO-TA/Blunt vector (Aidlab, Beijing, China) for dideoxy sequencing (Sangon, Shanghai, China). After sequence alignment, the cDNA sequences were submitted and deposited in NCBI under accession numbers PZ357124–PZ357127.

### 2.5. Developmental Expression Analysis of BtCDAs

To analyze the expression patterns of *BtCDAs*, approximately 200 eggs, 100 first- and second-instar nymphs, 50 third-instar nymphs, 40 fourth-instar nymphs, and 20 newly emerged (within 24 h post-eclosion) mixed-sex adults were collected. RNA extraction and cDNA synthesis were performed following the procedures described in Section 2.4. Reverse-transcription quantitative PCR (RT-qPCR) analysis was performed on an FQD-96A real-time PCR system (Bioer, Hangzhou, China) using a ChamQ Blue Universal SYBR qPCR Master Mix (Vazyme, Nanjing, China). All primers used for RT-qPCR are listed in Supplementary Table S1. The elongation factor 1-alpha (*EF1α*) gene (NCBI accession: EE600682) served as the reference gene for analyzing *BtCDA* expression levels across developmental stages. Each sample was analyzed in triplicate, and target gene expression levels were calculated using the 2^−ΔΔCt^ method [41].

### 2.6. dsRNA Synthesis and RNAi Experiment

Two 400-base pair (bp) DNA fragments of *btCDA1 and btCDA4* genes were selected as templates for in vitro dsRNA synthesis. For *BtCDA2a* and *BtCDA2b*, the two spliced mRNA isoforms of *BtCDA2*, a shared 400 bp sequence was used as dsRNA transcription template. A chemically synthesized (Sangon, Shanghai, China) 300 bp fusion construct, designated as *3CDA*, was used to target a 100 bp region from the three *B. tabaci CDA* paralogs, *BtCDA1*, *BtCDA2a/b*, and *BtCDA4*. For cloning of the 3CDA fragment and screening of recombinants, the primers T7CDA4-F and T7CDA2a/2b-R were used. Specific primers with 5’ flanking T7 promoter sequences are listed in Supplementary Table S1. dsRNA targeting the enhanced green fluorescent protein (EGFP) gene served as a negative control in the RNAi experiment. dsRNA was prepared using a T7 RNAi Transcription Kit (Vazyme, Nanjing, China). The quality of the dsRNA was verified by 1% agarose gel electrophoresis and assessed using a microvolume spectrophotometer.

In vitro transcription of dsRNA and subsequent RNAi experiments were conducted using 30 ng of dsRNA. To facilitate dsRNA delivery, a nanomaterial SPc, or star polycation, was used to package dsRNA [33,34]. The preparation of the SPc/dsRNA complex and application of dsRNA on *B. tabaci* have been described previously [15]. Briefly, 50 *B. tabaci* nymphs of similar size at the late third or early fourth instar stage were selected and placed on the abaxial surface of cotton leaves. A 10 nL dsRNA/SPc mixture (containing 3 μg/μL dsRNA and 3 μg/μL SPc) was dropped onto the notum of the nymphs using a Nanoliter2020 nanoliter injector (WPI, Sarasota, FL, USA). At 24, 48, and 72 h post-treatment, expression levels of *BtCDAs* were assessed by RT-qPCR. Additionally, 75 nymphs were selected per biological replicate to record the mortality rate and adult eclosion rate at 12 h intervals post-treatment. Three biological replicates were established for all dsRNA treatment groups.

To minimize potential off-target effects, all dsRNA sequences were searched against the *B. tabaci* transcriptome to exclude sequences with significant homology (at least 19 bp continuous matches) to non-target genes using the NCBI blastn tool (Version 2.12.0).

### 2.7. Construction of dsRNA Expression Vectors and Plant Transformation

Plant dsRNA expression vectors were constructed following the method described by Yan et al. [42]. Primers with 5′- flanking universal sequences (Supplementary Table S1) were designed to amplify target fragments. To construct a plant expression plasmid, 50 ng of purified PCR product, 200 ng of pRNAi-GG plasmid, 0.5 μL of *Bsa* I, 0.5 μL of T4 DNA ligase, 1 μL of T4 DNA ligase buffer (New England Biolabs, Ipswich, MA, USA), and sterile distilled water were mixed to make a final volume of 10 μL. The reaction mixture was incubated at 37 °C for 2 h, followed by 80 °C for 5 min. The resulting product was transformed into *Escherichia coli* DH5α competent cells. After verification by dideoxy-sequencing, the recombinant plasmids were introduced into *Agrobacterium tumefaciens* LBA4404 competent cells. Recombinant plasmids were introduced into tomato (*Solanum lycopersicum* cv. Micro-Tom) following the method of Park et al. [43].

### 2.8. Plant-Mediated RNAi

Adults were used for the feeding assay because they are easy to transfer and can actively feed on transgenic plants. For the feeding assay, approximately 100 newly emerged (1-day-old) adult whiteflies were released into each insect-proof cage (20 cm × 20 cm × 45 cm) containing one transgenic tomato plant. The whiteflies were allowed to feed on the plants for 3 days. After 3 days, surviving adults were collected for RNA extraction and RT-qPCR analysis (primers are listed in Supplementary Table S1). The dsEGFP-expressing tomato plants were used as the control. Three biological replicates were performed for each treatment.

### 2.9. Statistical Analysis

All experimental data were analyzed using GraphPad Prism 10.0 software (GraphPad Software Inc., San Diego, CA, USA). For developmental expression data, one-way ANOVA with Tukey’s HSD test was used for multiple comparisons. Gene expression levels following dsRNA treatment were analyzed by two-way ANOVA with Sidak’s multiple comparisons test. Mortality data after dsRNA treatment were analyzed by two-way ANOVA with Dunnett’s post hoc test. Eclosion rates were analyzed by Welch’s ANOVA with the Games–Howell post hoc test for multiple comparisons.

## 3. Results

### 3.1. Identification and Screening of BtCDAs

**Figure 1:**
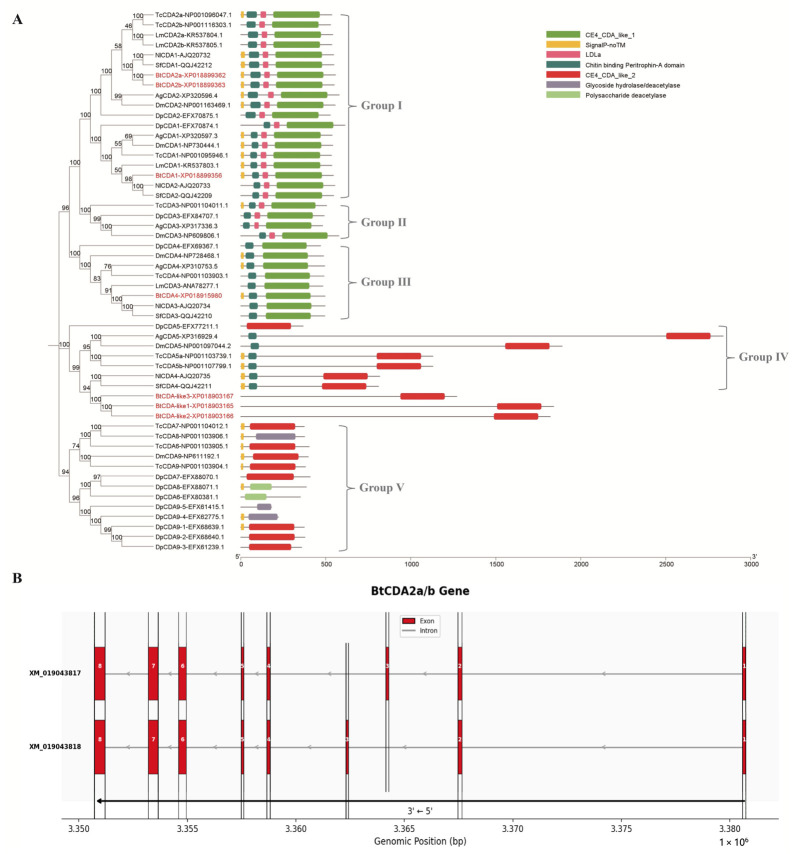
Phylogeny and sequence similarity of candidate BtCDAs. (**A**) Phylogenetic analyses and domain organization of insect chitin deacetylases (CDAs) from *Anopheles gambiae* (Ag), *Drosophila melanogaster* (Dm), *T. castaneum* (Tc), *Daphnia pulex* (Dp), *Locusta migratoria* (Lm), *N. lugens* (Nl), *S. furcifera* (Sf), and *B. tabaci* (Bt). The amino acid sequences of CDAs were classified into five groups (I–V). Protein names in red font indicate sequences derived from *Bemisia tabaci*. (**B**) Genomic sequence structure of two alternatively spliced *BtCDA2* genes. *BtCDA2a* and *BtCDA2b* use different exon 3, resulting in approximately 100 bp sequence divergence. Arrows indicate that *BtCDA2a/b* are transcribed in the right-to-left direction in the figure.

Using the HMMER3.4 package suite, seven candidate chitin deacetylases (CDAs) from the *B. tabaci* genome were identified, designated BtCDA1, BtCDA2a, BtCDA2b, BtCDA4, BtCDA-like1 (NCBI accession: XP_018903165), BtCDA-like2 (NCBI accession: XP_018903166), and BtCDA-like3 (NCBI accession: XP_018903167). Based on phylogenetic analysis and domain prediction (Figure 1A), BtCDA-like1, BtCDA-like2, and BtCDA-like3 were clustered into Group IV but lacked the chitin-binding domain (ChBD); consequently, these three genes were excluded from the *B. tabaci* CDA family. Finally, four proteins possessing the essential CDA domains were confirmed as CDA members in the *B. tabaci* genome (Table 1).

### 3.2. Molecular Characterization and Phylogenetic Analysis of BtCDAs

cDNA sequences of the four *BtCDA* genes were cloned and sequenced. The open reading frames (ORFs) were determined to be 1638, 1671, 1653, and 1488 bp in length, encoding proteins of 545, 556, 550, and 495 amino acid residues, respectively (Table 1). Subcellular localization prediction indicated that all four proteins are secreted into the extracellular matrix, consistent with previous studies on chitin deacetylases [44,45].

Phylogenetic analysis revealed that the red flour beetle, *T. castaneum*, possesses CDAs belonging to all five groups (Groups I–V) [31,32], whereas only Group I and Group III CDAs were identified in *B. tabaci (*Figure 1A). Specifically, BtCDA1, BtCDA2a, and BtCDA2b were classified into Group I, and BtCDA4 into Group III (Figure 1A). This distribution pattern differs from the discovery of Group I, III, and IV CDAs in other Hemipteran insects, like *N. lugens*, *Acyrthosiphon pisum*, and *S. furcifera* [28,29]. Notably, *BtCDA2a* and *BtCDA2b* are two alternatively spliced isoforms of *BtCDA2*, exhibiting 96.18% nucleotide sequence identity in their coding DNA sequence (CDS) regions (Figure 1B).

### 3.3. Developmental Expression Patterns of BtCDAs

**Figure 2:**
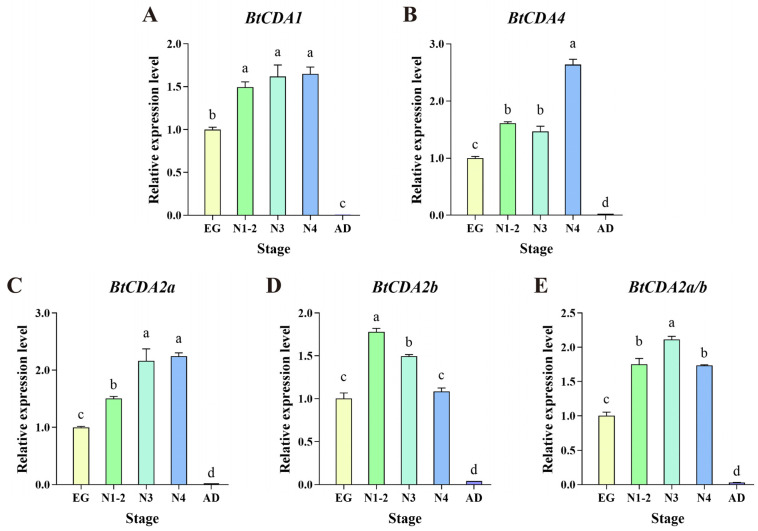
Expression of four *BtCDAs* at various developmental stages in *B. tabaci*. (**A**–**E**) Relative expression levels of *BtCDA1* (**A**), *BtCDA2a* (**B**), *BtCDA2b* (**C**), *BtCDA4* (**D**), and *BtCDA2a/b* (**E**) at Egg (EG), N1–N4 (first–fourth instar nymph), and adult (AD) stages. Different lowercase letters above the bars indicate significant differences in expression levels among developmental stages for a given gene (one-way ANOVA followed by Tukey’s HSD test, *p* < 0.05). Bars: ±standard error of the mean (SEM).

To investigate the expression profiles of *BtCDA* genes, RT-qPCR was performed to determine their transcript levels across different developmental stages of *B. tabaci* (Figure 2). Transcript levels of *BtCDA1*, *BtCDA2a*, and *BtCDA4* increased progressively during egg and nymphal development. In contrast, *BtCDA2b* expression declined from the first through fourth instar, suggesting the difference in gene function between *BtCDA2a* and *BtCDA2b*. Notably, all four genes showed very low expression levels in adults.

### 3.4. Functional Analysis of BtCDAs by RNAi

**Figure 3:**
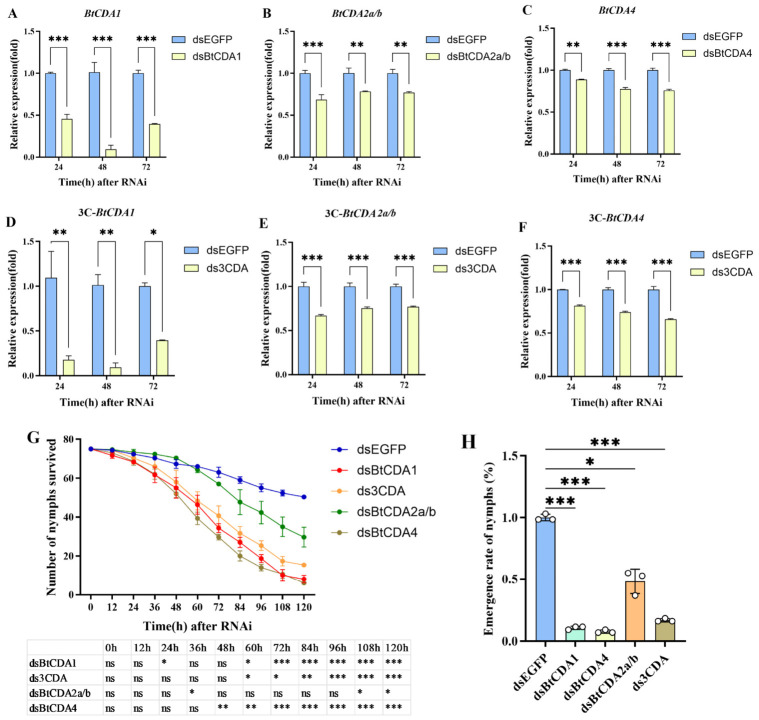
Silencing of *BtCDA* genes by SPc-delivered dsRNA in *B. tabaci*. (**A**–**C**) Relative expression levels of *BtCDA1*, *BtCDA2a/b*, and *BtCDA4* at 24, 48, and 72 h after treatment with dsRNA targeting the corresponding single gene. (**D**–**F**) Relative expression levels of *BtCDA1*, *BtCDA2a/b*, and *BtCDA4* at 24, 48, and 72 h after treatment with dsRNA targeting the fused *3CDA* gene. (**G**) Corrected mortality of fourth-instar nymphs after treatment with ds3CDA, dsBtCDA1, dsBtCDA2a/b, dsBtCDA4, or dsEGFP (control). Mortality was recorded every 12 h up to 120 h. (**H**) Relative emergence rate of adults after each dsRNA treatment. All values are shown as mean ± SEM. Asterisks indicate significant differences compared with the dsEGFP control (*ns*, *not significant*; *, p < 0.05; **, p < 0.01; ***, *p < 0.001*).

To evaluate the biological functions of chitin deacetylase genes in *B. tabaci*, we performed functional analysis of CDAs through SPc-mediated RNAi. At 72 h post-dsRNA treatment, the expression levels of all CDA genes were significantly downregulated compared with the control group treated with dsEGFP (Figure 3A–C). The expression levels of *BtCDA1*, *BtCDA2a/b*, and *BtCDA4* were reduced by approximately 60.7%, 23.2%, and 24.4%, respectively. Notably, for *BtCDA1* and *BtCDA4*, transcript levels at 72 h were slightly higher than at 48 h, showing a rebound of expression after the initial silencing. A similar temporal pattern has been observed in *S. furcifera* after dsRNA injection, where target gene expression at 72 h was higher than at 48 h [29]. Furthermore, *dsBtCDA4* treatment induced continuous and significant mortality in *B. tabaci* nymphs starting from 48 h post-treatment. dsBtCDA1 treatment caused continuous and significant mortality in *B. tabaci* nymphs starting from 60 h post-treatment. In contrast, continuous significant differences were only observed for dsBtCDA2a/b treatment starting from 108 h post-treatment. The corrected mortality rates of *B. tabaci* nymphs at 120 h were 84.1%, 88.1%, and 40.8% for dsBtCDA1, dsBtCDA4, and dsBtCDA2a/b treatments, respectively (Figure 3G). For dsRNA treatments targeting different CDA genes, the final relative eclosion rates were 11.5%, 7.5%, and 48.1% (dsBtCDA1, dsBtCDA4, and dsBtCDA2a/b, respectively) (Figure 3H).

For RNAi of the fusion gene *3CDA*, all three CDA genes exhibited downregulated expression, with *BtCDA1* showing the most prominent reduction. At 72 h post-ds3CDA treatment, the expression levels of *BtCDA1*, *BtCDA2a/b*, and *BtCDA4* decreased by approximately 60.7%, 23.1%, and 34.3%, respectively (Figure 3D–F). Compared with interference of single CDA genes, the fusion gene interference demonstrated more significant downregulation of gene expression. Furthermore, ds3CDA treatment caused continuous and significant mortality in *B. tabaci* nymphs starting from 60 h post-treatment. At 120 h, the corrected mortality rate of *B. tabaci* nymphs reached 69.5%, with a relative eclosion rate of 16.8% (Figure 3G,H).

### 3.5. Expression Levels of BtCDAs in B. tabaci Adults Feeding on Transgenic Tomato Plants

**Figure 4:**
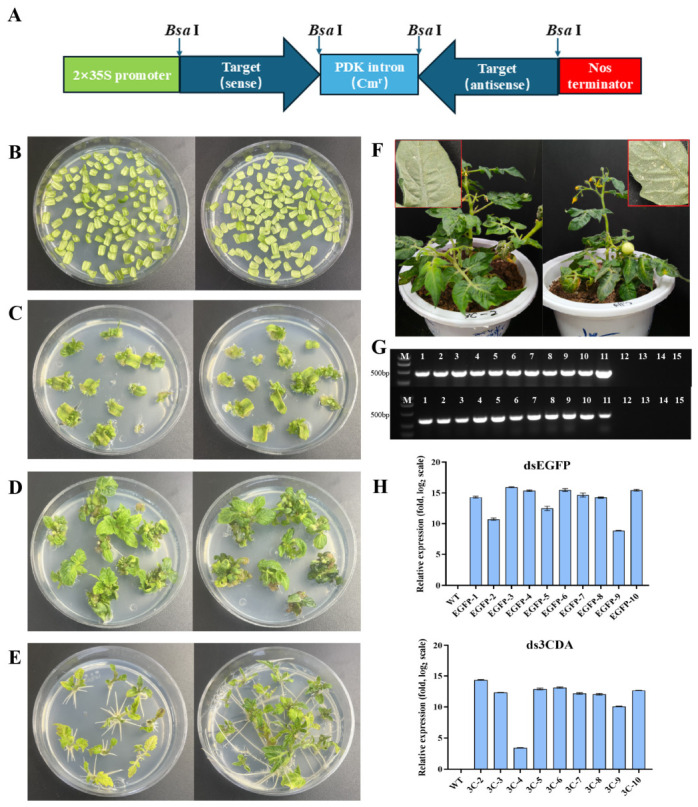
Construction and molecular characterization of transgenic tomato lines expressing dsRNA targeting *3CDA* or *EGFP*. (**A**) Schematic representation of the pRNAi-GG expression cassette used for tomato transformation. (**B**–**F**) Key steps of *Agrobacterium*-mediated tomato transformation: (**B**) co-cultivation of explants with *Agrobacterium*, (**C**) screening of transformed cells on medium containing antibiotics, (**D**) shoot differentiation, and (**E**) rooting of regenerated shoots. (**F**) Potted transgenic plants expressing ds3CDA and dsEFGP. Pests colonizing on the surface of the back of the leaf blades are shown in the red-boxed photos. (**G**) Genomic identification of transgenic lines by PCR: detection of dsEGFP (upper panel) and ds3CDA (lower panel) fragments. M, DNA marker; lanes 1–10 represent independent lines; lane 11 indicates positive plasmid control; and lanes 12–15 are wild-type control. (**H**) Relative expression levels of the dsRNA in transgenic lines detected by RT-qPCR. RT-qPCR data of Plant 3C-1 are missing due to the death of the plant prior to RT-qPCR analysis, potentially due to unanticipated experimental factors. For the same reason, during the release of whitefly adults to evaluate developmental and lethal effects, the majority of the transgenic plants withered and died prematurely prior to the completion of observation and statistical analyses.

**Figure 5:**
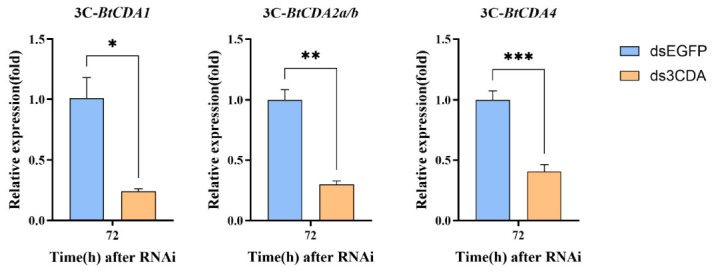
Expression levels of *BtCDA* genes in *B. tabaci* adults after feeding on transgenic tomato plants expressing ds3CDA. Whitefly adults were allowed to feed on transgenic tomato leaves for 3 days. Relative expression of *BtCDA1*, *BtCDA2a/b*, and *BtCDA4* was determined by RT-qPCR. The dsEGFP transgenic tomato line was used as the control. All values are shown as mean ± SEM. Asterisks indicate significant differences compared with the control (Welch’s *t*-test; *, p < 0.05; **, p < 0.01; ***, *p < 0.001*).

Transgenic tomato plants expressing dsRNAs of *3CDA* and *EGFP* were constructed via *Agrobacterium*-mediated transformation using the pRNAi-GG plasmid (Figure 4A) (Figure 4B–F). Based on PCR genotyping and RT-qPCR expression level analyses, 10 dsEGFP transgenic plants and 9 ds3CDA transgenic plants were obtained (Figure 4G,H). We selected high-expressing transgenic plants (EGFP-3, 4, 6 and 3CDA-2, 5, 6) for subsequent feeding experiments. Whiteflies were released on each of these lines separately, and the results were pooled as mean ± SEM. The results showed that after *B. tabaci* fed on transgenic plant leaves for 3 days, the expression levels of all BtCDA genes were significantly downregulated compared with those in whiteflies fed on dsEGFP tomato plants. The average expression levels of *BtCDA1*, *BtCDA2a/b*, and *BtCDA4* were 23.9%, 30.0%, and 40.5% of control levels, respectively (Figure 5). Three weeks after releasing adult *B. tabaci*, observation of the abaxial side of transgenic tomato leaves revealed fewer eggs, nymphs, and exuviae of *B. tabaci* on plants expressing ds3CDA compared to those expressing dsEGFP, indicating an inhibitory effect of dsCDA expression on the colonization of *B. tabaci*.

## 4. Discussion

Chitin deacetylases (CDAs) are key enzymes in chitin modification, playing indispensable roles in insect growth, molting, and metamorphosis [16,23]. In this study, we identified and cloned four CDA genes (*BtCDA1*, *BtCDA2a*, *BtCDA2b*, and *BtCDA4*) from the sweetpotato whitefly, *B. tabaci*. Phylogenetic analysis revealed that insect CDAs can be classified into five groups (Groups I–V) [31]. Among these, *BtCDA1* and *BtCDA2* belong to Group I and *BtCDA4* to Group III, while Groups II, IV, and V were not detected in *B. tabaci*. This is inconsistent with the findings in other hemipteran insects such as the brown planthopper (*N*. *lugens*), pea aphid (*A*. *pisum*), and the white-backed planthopper (*S*. *furcifera*). These hemipteran insects were reported to contain Group I, III, and IV CDAs [28,29], lacking Group II and Group V CDAs, which are commonly present in holometabolous insects such as the red flour beetle, *T*. *castaneum* [32]. Despite the absence of Group IV CDAs, three CDA-like proteins, which cluster with insect Group IV CDAs but lack the chitin-binding peritrophin-A domain, were present in *B. tabaci* (Figure 1A), suggesting an evolutionary divergence between *B. tabaci* and other hemipteran insects. The conserved reduction in the CDA families likely reflects an evolutionary adaptation to hemimetabolous development and piercing-sucking feeding habits in Hemiptera, characterized by the absence of a pupal stage and relatively simple peritrophic matrix structure in the midgut, thereby reducing functional requirements for certain CDA subtypes [28]. Notably, two highly homologous paralogs, BtCDA2a and BtCDA2b, exist in Group I CDA of *B. tabaci*, sharing 96.18% nucleotide sequence identity in their coding sequences. A similar scenario has been reported in *N. lugens*, where *CDA2* exhibits alternative splicing variants with differential expression across developmental stages [28]. This suggests that Group I CDAs may have undergone functional divergence in hemipteran insects, with different isoforms potentially assuming distinct physiological roles—some primarily responsible for cuticular chitin modification and others participating in the formation of specific structures, such as wings or joints, a phenomenon well-documented in *T. castaneum* and *Drosophila* [32,46]. The observed developmental expression patterns of *BtCDAs* can be well explained by their proposed physiological roles. The high transcription levels during the egg and nymph stages correlate with the active periods of chitin synthesis and remodeling required for embryonic development, hatching, and successive molts. Conversely, the extremely low expression in adults is expected, as the adult cuticle is largely sclerotized and no longer undergoes expansion, and the peritrophic matrix, once formed, may require less active chitin modification. This pattern further supports the notion that targeting *BtCDAs* with RNAi during the nymph stage, when these genes are most active, is a highly effective strategy for pest control [47].

Through SPc nanoparticle-mediated dsRNA delivery, this study systematically evaluated the biological functions of the four *BtCDA* genes. Topical application of dsRNA or nanocarrier-dsRNA complexes via droplets on the insect cuticle has been successfully demonstrated in several species, including *B. tabaci* [15], *Apolygus lucorum* [48], *S. frugiperda* [49], and *Dendroctonus valens* [50]. This simple, non-invasive strategy offers a practical means to partially mimic field-spraying conditions [15]. RNAi results showed that silencing *BtCDA4* and *BtCDA1* caused 84.1% and 88.1% corrected mortality in fourth instar nymphs of *B. tabaci*, respectively, whereas silencing *BtCDA2a/b* resulted in only 40.8% mortality. These findings are highly consistent with observations in *N. lugens* and *S. furcifera*: in *N. lugens*, RNAi of *NlCDA4* (Group III) caused over 95% mortality, with a stronger lethal effect than Group I CDAs [28]; in *S. furcifera*, silencing of *SfCDA4* similarly induced severe molting defects and high mortality, while the effect of *SfCDA2* was relatively mild [29]. Collectively, these results indicate that Group III CDA (CDA4) may play a more critical role than Group I CDA during the nymph-to-adult molting transition in hemipteran insects. This functional distinction may be attributed to the unique domain architecture of CDA4, which contains only a chitin-binding domain (CBD) and a CE4 catalytic domain but lacks the LDLa domain—a simplified structure that may confer distinct catalytic activity or substrate specificity [23,24]. Furthermore, the relatively low mortality observed upon *BtCDA2a/b* silencing in this study, combined with their high sequence similarity, suggests that these two genes may be functionally redundant, making it difficult to completely abolish their overall function with single dsRNA treatment. This partially explains why the fusion gene *3CDA* (simultaneously targeting *BtCDA1*, *BtCDA2a/b*, and *BtCDA4*) achieved more efficient gene silencing and higher mortality (69.5% corrected mortality at 120 h, with only 16.8% relative eclosion rate). The fusion gene strategy, which concatenates multiple target fragments into a single dsRNA molecule, enables simultaneous interference of multiple key genes, enhancing RNAi efficiency while potentially reducing the risk of pest resistance to single-target dsRNAs [51]. This multi-target synergistic interference strategy has demonstrated significant advantages in RNAi-based pest control research on hemipteran insects [51,52].

The SPc nanoparticle used in this study significantly enhanced dsRNA stability and delivery efficiency, achieving sustained gene silencing for up to 72 h. SPc is a specially designed amphiphilic cationic polymer that can self-assemble with dsRNA into nanocomplexes of approximately 100–200 nm through electrostatic interactions [34]. Nanocarriers effectively protect dsRNA from degradation by ribonucleases in insect hemolymph and gut [15,53]. In the green mirid bug (*A*. *lucorum*), SPc-mediated delivery of dsRNA targeting ecdysone receptor and trehalase genes effectively inhibited insect growth and development [48]. In the present study and our previous studies [15], SPc/dsRNA complexes were directly applied to the dorsal epidermis of fourth instar nymphs of *B. tabaci*, a simple practice causing minimal damage to the insects, overcoming the limitations of microinjection methods, which are tedious and unsuitable for large-scale application.

The SPc-mediated RNAi silencing efficiencies observed in this study range from 60.7% to 23.2% at 72 h. This is only a moderate level of gene silencing in comparison to reported SPc-mediated RNAi. For example, in our previous work, SPc-mediated RNAi of five other chitin metabolism-related genes led to target gene expression being decreased by over 70% at 72 h post RNAi [15]. Significant variation in gene silencing efficiency is a common feature of nanomaterial-mediated RNAi. Specifically, RNAi mediated by various nanomaterials targeting distinct genes has yielded gene silencing efficiencies ranging from 23% to 98% [54]. The relatively moderate knockdown levels in this study might be attributed to the specific target genes, the dsRNA delivery strategy, and the length of the dsRNA constructs [55,56], highlighting the critical need to optimize both target gene selection and dsRNA design.

In the present work, we further constructed transgenic tomato plants expressing ds3CDA and validated the feasibility of plant-mediated RNAi through feeding assays. Results showed that after adult whiteflies fed on transgenic tomato leaves for 3 days, the expression levels of *BtCDA1*, *BtCDA2a/b*, and *BtCDA4* decreased by 4.18-fold, 3.33-fold, and 2.47-fold compared to the control, respectively. Plant-mediated RNAi technology has achieved multiple successes in hemipteran pest control. Transgenic soybean plants expressing dsRNA targeting *Leguminivora glycinivorella* 18S ribosomal RNA showed enhanced resistance to this pest, achieving 35% mortality [57]. In *B. tabaci*, transgenic tobacco expressing dsRNA of *BtTPS1* and *BtTPS2* caused over 90% adult mortality and reduced fecundity [58]. Although the present study does not report data on developmental and lethal effects of the plant-expressed dsRNA against *B. tabaci*, our work nonetheless provides valuable evidence in establishing CDA genes as effective gene silencing targets for *B. tabaci* by plant-mediated RNAi. Further investigation will be conducted to analyze the abundance of B. tabaci eggs, nymphs, and exuviae on plants expressing ds3CDA or dsEGFP. Additionally, we will perform a long-term investigation to evaluate the gene silencing effects in *B. tabaci* nymphs rather than adults feeding on dsRNA-expressing transgenic plants.

In this study, both SPc nanoparticle-mediated delivery and transgenic plant-mediated expression of dsRNA were effective in silencing *BtCDA* genes. The SPc-mediated method resulted in a more potent gene silencing effect (e.g., decrease by 1.67-fold of *BtCDA1* at 72 h), likely due to the high concentration of dsRNA delivered directly to the target nymphs. However, this method is labor-intensive, cost-inefficient, and limited to laboratory applications. In contrast, plant-mediated RNAi, while achieving a moderate level of silencing (decrease by 2.47- to 4.18-fold compared to the control) in our short-term observation, offers a practical and scalable approach for field application. The continuous production of dsRNA by the plant provides sustained exposure, which could suppress pest populations over multiple generations. In this work, we used the pRNAi-GG vector, which produces hairpin RNA structures that are generally more stable than linear dsRNA. Additionally, the transgenic tomato plants showed consistent silencing effects over the experimental period, suggesting that dsRNA levels were sufficient to trigger RNAi in *B. tabaci*. Future optimization could include the design of artificial microRNA [59], co-expression of dsRNA-binding proteins [60], or the use of tissue-specific promoters to enhance dsRNA accumulation and stability in phloem, the primary feeding site of whiteflies [61].

In the future, further work will be undertaken to develop sprayable dsRNA formulations, explore synergistic effects of multiple RNAi targets, and systematically evaluate the environmental safety and resistance risks of RNAi technology to facilitate its transition from laboratory to field applications.

## 5. Conclusions

In conclusion, in this study, we identified four CDA members from the genome of the MEAM1 cryptic species of *B. tabaci*. Significant expression of the four *btCDAs* was detected in the egg and various nymphal stages. Both nanoparticle-mediated dsRNA delivery and transgenic plant-mediated RNAi strategies were proven to be feasible in *B. tabaci* control. These findings provide novel target genes and technical insights for RNAi-based green management of *B. tabaci*.

## Figures and Tables

**Table 1:** Characteristics of chitin deacetylase genes in *B. tabaci*.

Name	Protein	mRNA	Aa	Subcellular Localization	Hydrophilic/Hydrophobic	SP
BtCDA1	XP_018899356	XM_019043811	545	ECM	Hydrophilic	+
BtCDA2a	XP_018899362	XM_019043817	556	ECM	Hydrophilic	+
BtCDA2b	XP_018899363	XM_019043818	550	ECM	Hydrophilic	+
BtCDA4	XP_018915980	XM_019060435	495	ECM	Hydrophilic	+

Note: ECM indicates an extracellular matrix; SP indicates a signal peptide; + indicate presence, respectively.

## Data Availability

All data in this study, such as the gene entry number, are available on the NCBI website (National Center for Biotechnology Information).

## Supplementary Materials

The following supporting information can be downloaded at: https://www.mdpi.com/article/10.3390/insects17060628/s1, Table S1: Primers used in this study.

## References

[1] De Barro P.J., Liu S.S., Boykin L.M., Dinsdale A.B. (2011). *Bemisia tabaci*: A statement of species status. Annu. Rev. Entomol..

[2] Ning W., Shi X., Liu B., Pan H., Wei W., Zeng Y., Sun X., Xie W., Wang S., Wu Q. (2015). Transmission of tomato yellow leaf curl virus by *Bemisia tabaci* as affected by whitefly sex and biotype. Sci. Rep..

[3] Li Y., Mbata G.N., Punnuri S., Simmons A.M., Shapiro-Ilan D.I. (2021). *Bemisia tabaci* on vegetables in the southern United States: Incidence, impact, and management. Insects.

[4] Dinsdale A., Cook L., Riginos C., Buckley Y.M., De Barro P. (2010). Refined global analysis of *Bemisia tabaci* (Hemiptera: Sternorrhyncha: Aleyrodoidea: Aleyrodidae) mitochondrial Cytochrome Oxidase 1 to identify species level genetic boundaries. Ann. Entomol. Soc. Am..

[5] Boykin L.M., De Barro P.J. (2014). A practical guide to identifying members of the *Bemisia tabaci* species complex: And other morphologically identical species. Front. Ecol. Evol..

[6] Houndété T.A., Kétoh G.K., Hema O.S., Brévault T., Glitho I.A., Martin T. (2010). Insecticide resistance in field populations of *Bemisia tabaci* (Hemiptera: Aleyrodidae) in West Africa. Pest Manag. Sci..

[7] Pan H., Preisser E.L., Chu D., Wang S., Wu Q., Carriére Y., Zhou X., Zhang Y. (2015). Insecticides promote viral outbreaks by altering herbivore competition. Ecol. Appl..

[8] Abdullah N.M.M., Singh J., Sohal B.S. (2006). Behavioral hormoligosis in oviposition preference of *Bemisia tabaci* on cotton. Pestic. Biochem. Physiol..

[9] Zhang J., Khan S.A., Heckel D.G., Bock R. (2017). Next-Generation insect-resistant plants: RNAi-mediated crop protection. Trends Biotechnol..

[10] Baulcombe D. (2004). RNA silencing in plants. Nature.

[11] Novina C.D., Sharp P.A. (2004). The RNAi revolution. Nature.

[12] Geley S., Müller C. (2004). RNAi: Ancient mechanism with a promising future. Exp. Gerontol..

[13] Walker W.B., Allen M.L. (2011). RNA interference-mediated knockdown of IAP in *Lygus lineolaris* induces mortality in adult and pre-adult life stages. Entomol. Exp. Appl..

[14] Chen J., Zhang D., Yao Q., Zhang J., Dong X., Tian H., Chen J., Zhang W. (2010). Feeding-based RNA interference of a trehalose phosphate synthase gene in the brown planthopper, *Nilaparvata lugens*. Insect Mol. Biol..

[15] Kong D., Gu H., Gao Y., Hou Y., Li J. (2026). Nanomaterial-mediated RNAi targeting chitin metabolism genes in MEAM1 cryptic species of *Bemisia tabaci* (Hemiptera: Aleyrodidae). Insects.

[16] Zhu K.Y., Merzendorfer H., Zhang W., Zhang J., Muthukrishnan S. (2016). Biosynthesis, turnover, and functions of chitin in insects. Annu. Rev. Entomol..

[17] Merzendorfer H. (2011). The cellular basis of chitin synthesis in fungi and insects: Common principles and differences. Eur. J. Cell Biol..

[18] Moussian B. (2010). Recent advances in understanding mechanisms of insect cuticle differentiation. Insect Biochem. Mol. Biol..

[19] Merzendorfer H., Zimoch L. (2003). Chitin metabolism in insects: Structure, function and regulation of chitin synthases and chitinases. J. Exp. Biol..

[20] Arakane Y., Muthukrishnan S. (2010). Insect chitinase and chitinase-like proteins. Cell. Mol. Life Sci..

[21] Zhu Q., Arakane Y., Banerjee D., Beeman R.W., Kramer K.J., Muthukrishnan S. (2008). Domain organization and phylogenetic analysis of the chitinase-like family of proteins in three species of insects. Insect Biochem. Mol. Biol..

[22] Zhang M., Du M.-Y., Wang G.-X., Wang Z.-Y., Lu Y.-J. (2020). Identification, mRNA expression, and functional analysis of chitin synthase 2 gene in the rusty grain beetle, *Cryptolestes ferrugineus*. J. Stored Prod. Res..

[23] Liu X., Zhang J., Zhu K.Y. (2019). Chitin in arthropods: Biosynthesis, modification, and metabolism. Adv. Exp. Med. Biol..

[24] Liang B., Song W., Xing R., Liu S., Yu H., Li P. (2023). The source, activity influencing factors and biological activities for future development of chitin deacetylase. Carbohydr. Polym..

[25] Guo W., Li G., Pang Y., Wang P. (2005). A novel chitin-binding protein identified from the peritrophic membrane of the cabbage looper, *Trichoplusia ni*. Insect Biochem. Mol. Biol..

[26] Zhong X.W., Wang X.H., Tan X., Xia Q.Y., Xiang Z.H., Zhao P. (2014). Identification and molecular characterization of a chitin deacetylase from *Bombyx mori* peritrophic membrane. Int. J. Mol. Sci..

[27] Tetreau G., Cao X., Chen Y.R., Muthukrishnan S., Jiang H., Blissard G.W., Kanost M.R., Wang P. (2015). Overview of chitin metabolism enzymes in *Manduca sexta*: Identification, domain organization, phylogenetic analysis and gene expression. Insect Biochem. Mol. Biol..

[28] Xi Y., Pan P.L., Ye Y.X., Yu B., Zhang C.X. (2014). Chitin deacetylase family genes in the brown planthopper, *Nilaparvata lugens* (Hemiptera: Delphacidae). Insect Mol. Biol..

[29] Yang X.B., Zhou C., Gong M.F., Yang H., Long G.Y., Jin D.C. (2021). Identification and RNAi-based functional analysis of four chitin deacetylase genes in *Sogatella furcifera* (Hemiptera: Delphacidae). J. Insect Sci..

[30] Yu H.-Z., Liu M.-H., Wang X.-Y., Yang X., Wang W.-L., Geng L., Yu D., Liu X.-L., Liu G.-Y., Xu J.-P. (2016). Identification and expression profiles of chitin deacetylase genes in the rice leaf folder, *Cnaphalocrocis medinalis*. J. Asia Pac. Entomol..

[31] Dixit R., Arakane Y., Specht C.A., Richard C., Kramer K.J., Beeman R.W., Muthukrishnan S. (2008). Domain organization and phylogenetic analysis of proteins from the chitin deacetylase gene family of *Tribolium castaneum* and three other species of insects. Insect Biochem. Mol. Biol..

[32] Arakane Y., Dixit R., Begum K., Park Y., Specht C.A., Merzendorfer H., Kramer K.J., Muthukrishnan S., Beeman R.W. (2009). Analysis of functions of the chitin deacetylase gene family in *Tribolium castaneum*. Insect Biochem. Mol. Biol..

[33] Li J.H., Qian J., Xu Y.Y., Yan S., Shen J., Yin M.Z. (2019). A facile-synthesized star polycation constructed as a highly efficient gene vector in pest management. ACS Sustain. Chem. Eng..

[34] Ma Z., Zheng Y., Chao Z., Chen H., Zhang Y., Yin M., Shen J., Yan S. (2022). Visualization of the process of a nanocarrier-mediated gene delivery: Stabilization, endocytosis and endosomal escape of genes for intracellular spreading. J. Nanobiotechnol..

[35] Zhang L.P., Zhang Y.J., Zhang W.J., Wu Q.J., Xu B.Y., Chu D. (2005). Analysis of genetic diversity among different geographical populations and determination of biotypes of *Bemisia tabaci* in China. J. Appl. Entomol..

[36] Jones P., Binns D., Chang H.-Y., Fraser M., Li W., McAnulla C., McWilliam H., Maslen J., Mitchell A., Nuka G. (2014). InterProScan 5: Genome-scale protein function classification. Bioinformatics.

[37] Katoh K., Standley D.M. (2013). MAFFT multiple sequence alignment software version 7: Improvements in performance and usability. Mol. Biol. Evol..

[38] Eddy S.R. (2011). Accelerated profile hmm searches. PLoS Comput. Biol..

[39] Tamura K., Stecher G., Kumar S. (2021). MEGA11: Molecular evolutionary genetics analysis version 11. Mol. Biol. Evol..

[40] Chen C., Chen H., Zhang Y., Thomas H.R., Frank M.H., He Y., Xia R. (2020). TBtools: An integrative toolkit developed for interactive analyses of big biological data. Mol. Plant.

[41] Livak K.J., Schmittgen T.D. (2001). Analysis of relative gene expression data using real-time quantitative PCR and the 2^−ΔΔCt^ method. Methods.

[42] Yan P., Shen W., Gao X., Li X., Zhou P., Duan J. (2012). High-throughput construction of intron-containing hairpin RNA vectors for RNAi in plants. PLoS ONE.

[43] Park S.H., Morris J.L., Park J.E., Hirschi K.D., Smith R.H. (2003). Efficient and genotype-independent Agrobacterium-mediated tomato transformation. J. Plant Physiol..

[44] Tsigos I., Martinou A., Kafetzopoulos D., Bouriotis V. (2000). Chitin deacetylases: New, versatile tools in biotechnology. Trends Biotechnol..

[45] Grifoll-Romero L., Pascual S., Aragunde H., Biarnés X., Planas A. (2018). Chitin deacetylases: Structures, specificities, and biotech applications. Polymers.

[46] Luschnig S., Bätz T., Armbruster K., Krasnow M.A. (2006). Serpentine and vermiform encode matrix proteins with chitin binding and deacetylation domains that limit tracheal tube length in Drosophila. Curr. Biol..

[47] Doucet D., Retnakaran A., Dhadialla T.S. (2012). Insect Chitin: Metabolism, Genomics and Pest Management. Advances in Insect Physiology, Vol 43: Insect Growth Disruptors.

[48] Qiao H., Jiang Q., Zhao J., Xiao L., Zhu-Salzman K., Xu D., Xu G., Shen J., Gu A., Hao D. (2025). Nano-delivery platform with strong protection and efficient delivery: Preparation of self-assembled RNA pesticide with dual RNAi targets against *Apolygus lucorum*. J. Nanobiotechnol..

[49] Guo S., Li Z., Zhao X., Zhang D., Ayra-Pardo C., Kan Y., Li D. (2025). Additive insecticidal effects of chitosan/dsRNA nanoparticles targeting V-ATPaseD and emamectin benzoate–lufenuron formulations against *Spodoptera frugiperda* (J.E. Smith) (Lepidoptera: Noctuidae). Insects.

[50] Cao Q., Sun Y., Kong D., Han J., Wei J., Li J. (2026). Potential of RNAi targeting Juvenile Hormone Acid Methyltransferase (JHAMT) for controlling *Dendroctonus valens* LeConte (Coleoptera: Scolytidae). Forests.

[51] Xu Q.Q., Shang F., Feng S.Y., Xie Q.P., Zhang W., Wang Z.G., Wang J.J. (2024). Design the fusion double-strand RNAs to control two global sap-sucking pests. Pestic. Biochem. Physiol..

[52] Wang Z.G., Qin C.Y., Chen Y., Yu X.Y., Chen R.Y., Niu J., Wang J.J. (2024). Fusion dsRNA designs incorporating multiple target sequences can enhance the aphid control capacity of an RNAi-based strategy. Pest Manag. Sci..

[53] Christiaens O., Whyard S., Vélez A.M., Smagghe G. (2020). Double-stranded RNA technology to control insect pests: Current status and challenges. Front. Plant Sci..

[54] Liu J.S., He Q.Y., Lin X.F., Smagghe G. (2025). Recent progress in nanoparticle-mediated RNA interference in insects: Unveiling new frontiers in pest control. J. Insect Physiol..

[55] Liu S.S., Jaouannet M., Dempsey D.M.A., Imani J., Coustau C., Kogel K.H. (2020). RNA-based technologies for insect control in plant production. Biotechnol. Adv..

[56] San Miguel K., Scott J.G. (2016). The next generation of insecticides: dsRNA is stable as a foliar-applied insecticide. Pest Manag. Sci..

[57] Wang Z., Li T., Ni H., Wang G., Liu X., Cao Y., Li W., Meng F. (2018). Transgenic soybean plants expressing *Spb18S* dsRNA exhibit enhanced resistance to the soybean pod borer *Leguminivora glycinivorella* (Lepidoptera: Olethreutidae). Arch. Insect Biochem. Physiol..

[58] Gong C., Yang Z., Hu Y., Wu Q., Wang S., Guo Z., Zhang Y. (2022). Silencing of the BtTPS genes by transgenic plant-mediated RNAi to control *Bemisia tabaci* MED. Pest Manag. Sci..

[59] Bao W., Li A., Zhang Y., Diao P., Zhao Q., Yan T., Zhou Z., Duan H., Li X., Wuriyanghan H. (2021). Improvement of host-induced gene silencing efficiency via polycistronic-tRNA-amiR expression for multiple target genes and characterization of RNAi mechanism in *Mythimna separata*. Plant Biotechnol. J..

[60] Gillet F.X., Garcia R.A., Macedo L.L.P., Albuquerque E.V.S., Silva M.C.M., Grossi-de-Sa M.F. (2017). Investigating engineered ribonucleoprotein particles to improve oral RNAi delivery in crop insect pests. Front. Physiol..

[61] Javaid S., Amin I., Jander G., Mukhtar Z., Saeed N.A., Mansoor S. (2016). A transgenic approach to control hemipteran insects by expressing insecticidal genes under phloem-specific promoters. Sci. Rep..

