# Supplementary material for: Identification and RNA Interference-Based Functional Analysis of Chitin Deacetylase Genes in Bemisia tabaci
Source: Insects. 2026 Jun 15;17(6):628. doi: 10.3390/insects17060628 (PMC13302401; doi:10.3390/insects17060628)
Supplement: Supplementary file 1 [file insects-17-00628-s001.zip › insects-4333384-supplementary.pdf]

Table S1. Primers used in this study.

PCR reactions	Primer name	Primer sequence（5'-3'）
PCR	BtCDA1-F	CAGCCGACCACTGTAGTGAA
	BtCDA1-R	CTAGTCGGATTCGGTTCGGG
	BtCDA2a/b-F	TGGTTCACAGTTAGTCCCCG
	BtCDA2a/b-R	CTTCGGAGGGAACGATGAGG
	BtCDA2bm-R	TGTTTTATGCCAGCTTTAGTAC
	BtCDA4-F	AGAAAGTCCAAACTCCCCGC
	BtCDA4-R	GCTTTTGACGATTTTGTGGGAGA
RT-qPCR	qBtCDA1-F	CGTATTGTGTTTGGCTTTAC
	qBtCDA-R	ACTCCTCTTGTCTCTTAACT
	qBtCDA2a-F	AGATTGCCGAGATGTGGTCAG
	qBtCDA2a-R	TATATCGAAAGCGAGCCCGTT
	qBtCDA2b-F	CGTGTGATTGGAAGGGTAAAGTG
	qBtCDA2b-R	CGGTTCGTCAGTCTTGAGGAT
	qBtCDA2a/b-F	AACGAGAAGAACGATGTCTA
	qBtCDA2a/b-R	TGGAAATCTCTCAAGGAACT
	qBtCDA4-F	CTACCCATACTACCAGAAAG
	qBtCDA4-R	CTTGTTGAAGTGAGATTGTC
	QEF1α-F	GTAAGGACGGTAATGCTGAAGG
	QEF1α-R	TAGACATCTGGAGTGGCAATCG
dsRNA synthesis	T7-EGFP-F	TAATACGACTCACTATAGGGTACGGCGTGCAGTGCTTCA
	T7-EGFP-R	TAATACGACTCACTATAGGGGGTGCTCAGGTAGTGGTTGTC
	T7CDA1-F	TAATACGACTCACTATAGGGACACAATGAGGACGAGCACTT
	T7CDA1-R	TAATACGACTCACTATAGGGGAGGATGTTGGAGCAGGAGTC
	T7CDA2a/b-F	TAATACGACTCACTATAGGGTCTCGCTGACGCACAAGGA
	T7CDA2a/2b-R	TAATACGACTCACTATAGGGGGATGTTGGAGCAGGAGTCG
	T7CDA4-F	TAATACGACTCACTATAGGGACAGACAACGCAGTGAAGTGC
	T7CDA4-R	TAATACGACTCACTATAGGGAACAATGTCGCTTCTGGAGATG
dsRNA expression vector	G-EGFP-F	ACCAGGTCTCAGGAGTACGGCGTGCAGTGCTTCA
	G-EGFP-R	ACCAGGTCTCATCGTGGTGCTCAGGTAGTGGTTGTC
	G-3CDA-F	ACCAGGTCTCAGGAGACAGACAACGCAGTGAAGTGC
	G-3CDA-R	ACCAGGTCTCATCGTAACAATGTCGCTTCTGGAGATG
	P21	ACCATTTACGAACGATAGCC
	P22	GTAAAACGACGGCCAGTG
	P23	CGAATCTCAAGCAATCAAGC
	P24	CATTTTAGCTTCCTTAGCTCC
	P25	CATTTGGATTGATTACAGTTGG
	qEGFP-F	GGACGACGGCAACTACAAGA
	qEGFP-R	AAGTCGATGCCCTTCAGCTC
	qEXP-F	ACGGATTACGGTGGAGATGC
	qEXP-R	CCGGAGAATAGCATCCGGTC
	qIntro-F	TTCTTGCCCGCCTGATGAAT

qIntro-R	ACCGTAACACGCCACATCTT
